# Swine production: how sustainable is sustainability?

**DOI:** 10.1093/af/vfac085

**Published:** 2022-12-14

**Authors:** C E Vonderohe, L A Brizgys, J A Richert, J S Radcliffe

**Affiliations:** USDA-ARS Children’s Nutrition Research Center, Pediatrics, Gastroenterology & Nutrition, Baylor College of Medicine, Houston, TX 77030, USA; Department of Animal Sciences, Purdue University, West Lafayette, IN 47907, USA; Department of Animal Sciences, Purdue University, West Lafayette, IN 47907, USA; Department of Animal Sciences, Purdue University, West Lafayette, IN 47907, USA

**Keywords:** economics, environment, health, society, sustainability, swine

**Implications**

- Swine production is one piece of agriculture and agriculture is one piece of the planetary sustainability puzzle. Often boxes get drawn around various pieces of sustainability, but it is important to realize that sustainability of one box does not insure system-wide sustainability.
- To be sustainable, the swine industry must increase efficiency in an environmentally friendly and economically viable manner while maintaining excellent animal health and welfare, using societally acceptable production practices.
- Societal demands and concepts of sustainability may be counter-to what the science states. Swine production cannot move forward without societal approval, and this knowledge gap must be addressed.

## Introduction

Sustainability is a word that has seen increasing use in recent years. Unfortunately, the word is used differently in different situations, with different social and scientific connotations, often without definition or context. Although use of the word has become increasing popular within animal agriculture, “sustainable” agriculture is by no means a new concept. What is new or at least omnipresent is the modern-day societal pressure for agriculture to not only be sustainable but to prove that it is “sustainable.” The use of the term in the absence of a common definition or context, prompts the question what does sustainability mean, and the more important question, is sustainability sustainable? This paper will focus on sustainability relative to swine production. Even though, we are specifically addressing sustainability in swine production, it is important to remember that swine production is only one piece of animal agriculture sustainability puzzle, and animal agriculture is only one piece of global sustainability puzzle. The objective of this paper is not creation of a sustainable agriculture blueprint, but rather to pose questions and identify challenges that need to be addressed for the industry to eventually answer.

## Defining Sustainability

Start a classroom discussion by asking students in our field (animal science) to define sustainability, rarely will they come up with an overarching cohesive definition, rather, each student will focus on one element of the whole: the environment, animal health, or feeding the world. If we take a brief look at published definitions of sustainability, confusion is warranted. The Merriam-Webster dictionary (2022) defines sustainability as

1) Capable of being sustained
2) Of, relating to, or being a method of harvesting or using a resource so that the resource is not depleted or permanently damaged

The first definition from Merriam-Webster would have earned the authors a C- on a vocabulary assignment in middle school for using the root word in the definition. The second definition though overly simplistic in the scheme of animal agriculture, begins to apply to our current and future status in animal agriculture. In fact, the United States legal definition (U.S. Code Title 7, Section 3103; Sidebar 1) of sustainable agriculture aligns well with Merriam-Webster’s second definition.

For the purpose of simplicity, we are going to start with the student approach of breaking sustainability into parts and we will specifically reference the legal definition of agricultural sustainability presented in Sidebar 1 with a goal throughout the article of putting each part into the perspective of the whole. Note the legal definition has more than 4 parts, but we have combined all of the parts of the legal definition into the 4 discussed in this paper. Specifically, we will reference the following four parts of sustainability in swine production:

1) Environmental
2) Economic
3) Societal
4) Health/well-being of pigs and people

Please note our order in no way ranks one part of sustainability above another.

**Sidebar 1**

The United States (U.S. Code Title 7, Section 3103) defines sustainable agriculture as “an integrated system of plant and animal production practices having a site-specific application that will over the long-term:

- Satisfy human food and fiber needs.
- Enhance environmental quality and the natural resource base upon which the agriculture economy depends.
- Make the most efficient use of nonrenewable resources and on-farm resources and integrate, where appropriate, natural biological cycles and controls.
- Sustain the economic viability of farm operations.
- Enhance the quality of life for farmers and society as a whole.”

## Environmental Sustainability

The Environmental Protection Agency of the United States (EPA) states that “sustainability is based on a simple principle: Everything that we need for our survival and well-being depends, either directly or indirectly, on our natural environment. To pursue sustainability is to create and maintain the conditions under which humans and nature can exist in productive harmony to support present and future generations.” (National Research Council, 2011). Even though the EPA’s statement limits the conversation on sustainability to environment and that limitation is counter to our argument that agricultural sustainability and sustainability as a whole must be addressed in a broader context, it gives us a good starting place to discuss the environmental part of sustainability. Environmental sustainability of swine production can be distilled down to 3 major areas: Energy, Water, and Nutrient Balance.

Energy is used for crop production, feed manufacturing, feed distribution, lighting, heating, ventilation, and transportation of crops, feed, pigs, and manure. Electricity generation and heating are heavily reliant on fossil fuels in addition to the direct use of fuel to power vehicles for transportation and crop production. Using a Life Cycle Analysis (LCA) of farms the use of fossil fuel derived energy was estimated to be 10.6 MJ per kg of pig liveweight in the Midwestern region of the United States (Tallaksen et al., 2020). In addition, pigs consume large quantities of caloric energy for maintenance, growth, and reproduction and there is increasing global competition for energy resources.

Water is often overlooked because it is a renewable resource, but there are many regions of the world using water faster than it can be replenished, which clearly is not sustainable. In addition, contamination/pollution of water from swine production is a concern if nutrients in manure are mismanaged. While water is abundant on earth, potable water is not necessarily abundant. Only 0.5% of water is available as freshwater (Patience, 2012) with 98% of all fresh water located underground in aquifers (de Moel et al. 2006).

**Figure 1.**
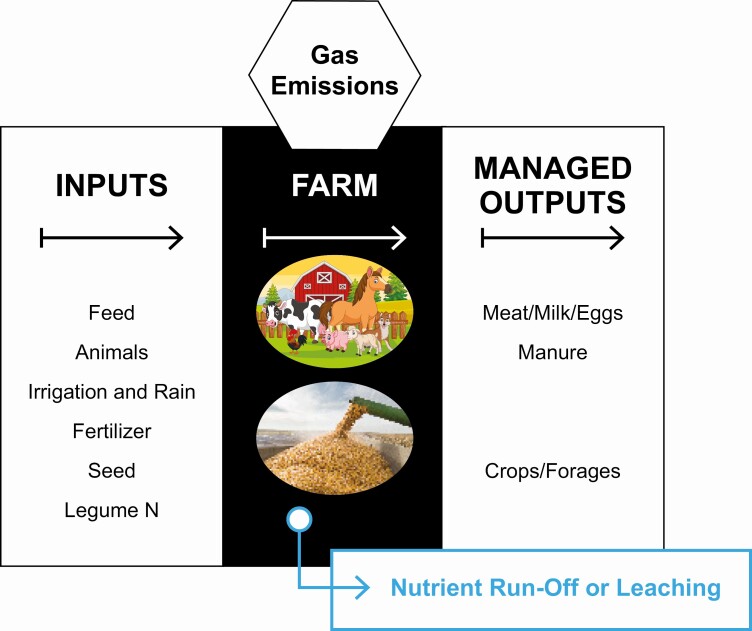
Nutrient flux onto and off of the farm.

Nutrient balance is a broad term used to describe the inputs and outputs of nutrients from swine production. The inputs and outputs change depending on whether we draw the environmental box around the pig, the farm, the swine industry as a whole or agriculture as a whole. To focus the discussion in this paper, we will discuss the flux of nutrients onto and off of the farm (Figure 1). Nutrients may enter the farm in the form of feed, animals, irrigation and rainwater, fertilizer, seed, and as atmospheric nitrogen trapped/fixed by legumes. Nutrients may leave the farm in animal products (meat, milk, fiber, and eggs), crops, and manure, all of which need to be managed. However, unmanaged nutrient losses can also occur via nutrient run-off or leaching or as gaseous emissions. Unmanaged nutrient losses cause the greatest concern. To reduce the environmental impact of swine production across these 3 areas, the swine industry has used genetic selection, enhanced/precision nutrition, altered management, and building/mechanical advancements.

### Impact of genetics on environmental sustainability of swine production

Traditional swine breeding programs have evolved over time to reflect changes in consumer demand, with the most recent public demand focusing on enhanced environmental sustainability and improvements in porcine welfare (Patience, 2015). Genetic selection of animals to increase environmental sustainability has rapidly gained popularity over the last decade, as many of the goals of sustainability (reduced economic and nutritive inputs) correspond with positive economic practices in swine production. Pigs can be genetically selected for an unlimited number of traits that may decrease environmental impact of their production. Obvious traits such as feed efficiency and rate of gain, and less obvious traits like positive maternal behaviors and improved reproductive efficiency, fundamentally have the same mechanism of increasing environmental sustainability, by reducing nutrient output. Although there is more and more research detailing the importance of selection of an infinitive number of traits on enhancing environmental sustainability, increasing feed efficiency is still the most utilized (Patience, 2015; Mote and Rothschild, 2020). Selection for feed efficiency supports increased production levels with reduced resources. Thereby reducing the energy needed to produce feed and decreasing nutrients in manure. Specific to the environment, selection for increased feed efficiency in pigs has also resulted in lower nitrogen and phosphorus excretion in growing pigs and creation of pigs that emit less greenhouse gases (Soleimani and Gilbert, 2021).

However, as we become more and more aggressive in selection for growth rate and feed efficiency, a new question emerges: at what point does selection affect the animal and result in negative consequence for the robustness of the animal? Put more simply, at what point have we increased growth rate and feed efficiency so far that our animals are growing faster than their anatomy can support? In addition to using genetic selection to enhance feed efficiency, several other production efficiency traits may yield decreased input of resources, and concomitant decreases in unmanaged nutrient losses, resulting in an overall increase in environmental sustainability of the production system (Patience, 2015). For instance, selection on sow performance and reproductive efficiency can result in fewer sows in a herd with a greater per sow output (Kemp, 2018). Selecting for more robust/healthier pigs or for sow longevity leads to more adaptable pigs that live longer, more productive lives, again reducing the total number of animals required to produce the current amount of pork, and enhancing environmental sustainability of pork production (Kemp, 2018). New data integration tools that assess the interactions between nutrition and genetics have done a great deal to both identify and integrate candidate traits into selection systems that will improve growth, health, welfare, and sustainability (Culbertson, 2017). Other emerging genetic tools, such as whole genome analysis, metagenomics, and gene editing are improving incorporation of traits important to sustainable pork production (Kemp, 2018).

However, beyond genetic selection, more rapid enhancements in genetic progress are being met with increasing societal reluctance. The Enviropig pig was a transgenic pig developed at the University of Guelph in the late 1990s that had dramatically reduced phosphorus excretion. While this pig represented a way to reduce the environmental footprint of swine production, no country ever approved human consumption of this transgenic pig and therefore the project was discontinued. More recently, researchers at the University of Missouri created a gene edited line of pigs that are resistant to Porcine Reproductive and Respiratory Syndrome (PRRSv) virus. The PRRS virus causes poor reproductive performance, reduced growth, and feed efficiency, and results in increased mortality. The impact of PRRS is estimated to cost North American swine farmers in excess of 650 million U.S. dollars annually. It has yet to be determined if society will accept a gene edited pig into the food chain, but genetically modified salmon and pigs have now been approved for consumption by the U.S. Food and Drug Administration. AquaAdvantage salmon were approved by the U.S. FDA for consumption in 2015. These fish have been genetically modified with a recombinant DNA construct to produce more growth hormone and as a result reach market weight approximately twice as fast as their nongenetically modified counterparts. In 2020, the U.S. FDA approved the GalSafe pig for human consumption and for medical use. This line of pigs has been genetically modified to not produce the cell surface sugar alpha-gal which can be a severe allergen.

### Impact of nutrition on environmental sustainability of swine production

Swine production and the push to optimize nutrient utilization into and out of the pig were the fundamental primers for the creation of the field of environmental nutrition. The main environmental impacts of pig production result from feed manufacturing, manure excretion, and gaseous emissions from animals and manure storage (Dourmad and Jondreville, 2007; Opio et al., 2013; Mackenzie et al., 2016). The environmental impact of swine production has been reduced through precision feeding (Gauthier et al., 2022) and dietary strategies to increase nutrient utilization and decrease nutrient excretion (Vonderohe et al., 2022), while attempts are made to capture excreted nutrients for further use. The ultimate goal is that everything that goes into the pig is used and if it comes out of the pig that it can be captured and used! Realistically, reducing unmanaged nutrient losses to zero is not achievable, but it is the goal.

**Figure 2.**
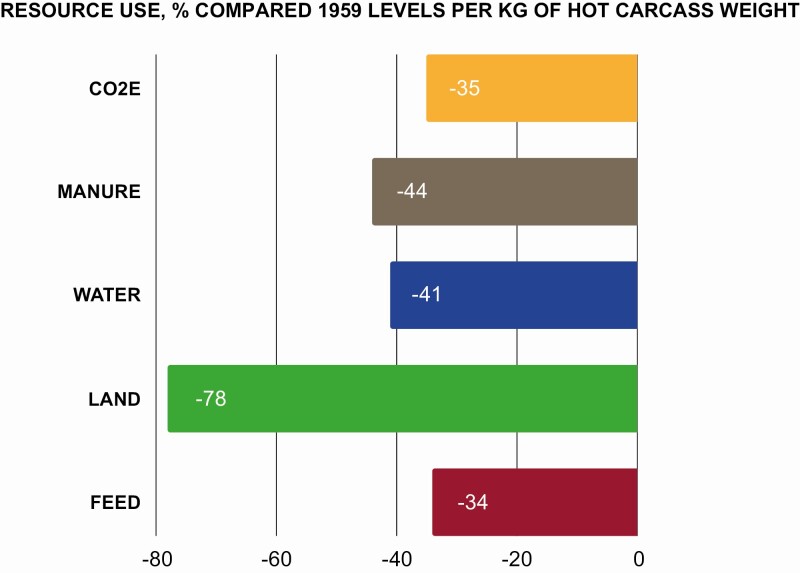
Improvements in the efficiency of pork production from 1969 to 2009 (Adapted from Boyd and Cady, 2012).

Boyd and Caty (2012) illustrated the improvements made by the swine industry when comparing U.S. swine production in 1959 with swine production in 2009 and reported that per kilogram of hot carcass weight (Figure 2):

- Manure volume has been decreased by 44%
- Water usage has been reduced by 41%
- Feed consumption has been decreased by 34%
- Emissions of greenhouse gases (CO_2_ equivalents) have been reduced by 35%

Taken at its most optimistic, the environmental impact of producing a single pork chop has been reduced by more than a third over the last 50 years. However, these numbers are often usurped by the increase in global population coupled with increased consumer demand for animal protein, which has resulted in a doubling of the total amount of pork produced from 1959 to 2009. As a result, while the environmental footprint of the industry was reduced per unit of pork produced, the total environmental footprint of pork production grew.

Improving feed efficiency has, and continues to be, a focus of nutrition in swine production, but many efforts in this area have become more specialized focusing on the efficiency of individual nutrient utilization. This has been achieved by a better understanding of nutrient requirements and the availability of nutrients within ingredients. Ultimately, this improved understanding has led to strategies to enhance nutrient availability from ingredients; for example dietary enzyme addition. Strategies to improve feed/nutrient utilization by the pig have also resulted in a reduced environmental footprint, because improvements in the ability of the pig to convert feed to product results in more nutrients staying in the pig and fewer nutrients being excreted.

However, while many advances have been made, there are increasing challenges to efficient feeding of the pig, and many of these challenges are a result of inter-industry competition for feed ingredients. For example, the use of corn for ethanol production has been driven in the United States by governmental policies, incentives, and mandates. The production of corn ethanol involves a process in which a variable amount of oil is extracted from corn and the majority of the carbohydrates are fermented to produce ethanol, leaving the fiber, mineral, and protein contents of corn in a product known as Distillers’ Dried Grains with Solubles (DDGS). Animal feed is the primary use for all DDGS but using DDGS in feed may adversely impact the sustainability of animal production. DDGS has a greater indigestible fiber content compared with corn, which leads to increased dry matter excretion. Additionally, the amino acid content of DDGS is more variable than other feed ingredients typically used in swine diets. The imbalance in amino acids (building blocks of protein) results in increased N excretion and ammonia emissions (Vonderohe et al., 2022). This example is not meant to pick on ethanol production per se, but the industry is illustrative of the fact that decisions made outside of swine production can often have widespread effects within the swine industry. That being said, in the ethanol expansion seen in the early 2000s, one of the other options discussed for DDGS was simply to landfill it. So, while feeding DDGS to livestock results in an increased environmental footprint of animal agriculture, it is less of an environmental footprint than if DDGS were simply disposed of in landfills.

### Impact of management and engineering strategies on environmental sustainability of swine production

The world swine population produces approximately 1.7 billion tonnes of liquid manure annually (Makara and Kowalski, 2018). Swine manure has a tremendous value as a natural fertilizer for crop production but must be managed properly to minimize greenhouse gas (GHG) emissions and nutrient run-off and leaching. The use of the phytase enzyme in swine diets has resulted in a more optimal ratio of nitrogen to phosphorus in manure (Sidebar 2). This coupled with improved manure application methods has resulted in more nutrients remaining in the soil for crop utilization and less build-up of nutrients as the ratio of nutrients in the soil more accurately matches the needs of crop production.

The major greenhouse gas emissions from swine production include ammonia (NH_3_), methane (CH_4_), and nitric oxide (N_2_O; Wang et al., 2017). Manure management is a major source of GHG emissions, contributing about 18% of all livestock GHG emissions (Dennehy et al. 2017). There are a variety of manure processing methods that can result in reduced emissions, but many of which are challenging because of the large amount of water added to swine manure. Switching from a liquid manure system to a liquid–solid separation system reduced GHG emissions by 65%, and NH_3_ emissions by 78% (Wang et al. 2017). Maraseni and Maroulis (2008) reported that anaerobic digestion could reduce CH_4_ emissions by 60%–85%. Ultimately, it will not be possible to have zero gas emissions from pigs and their manure. Therefore, it may be necessary to capture nutrients in emitted gases. Technologies already exist to do this today, and some are quite simple and relatively inexpensive. For example, biofilters, which can be as simple as passing outgoing air through a pile of wood chips are quite effective at reducing greenhouse gas emissions (Van Der Heyden, 2015). There are however tradeoffs; to push air through a filter of any kind requires a larger fan motor, which will use more electricity (Shang, 2022).

**Sidebar 2**

Phytase is an enzyme that catalyzes the release of phytate bound P. Phytate is the major storage form of P in plants and is poorly digested by the pig without the addition of the phytase enzyme to the diet. The net result is that pigs can be fed less total P, are able to more efficiently utilize, plant derived P, and excrete significantly less P.

### Economic sustainability

Unlike the complicated science involved in balancing input and outputs to produce pork in a more environmentally sustainable manner, it is fairly easy to understand economic sustainability: we must balance expenses and income (well actually ensure a profit) for any system to be economically sustainable. However, economic sustainability and environmental sustainability are often in conflict. In an extreme oversimplification, we must have enough money in our checking account to cover expenses. How much additional cost can this system absorb and be maintained or profitable? How much will people pay for pork? Can we produce pork (or other animal proteins) in a sustainable manner and remain economically viable?

Livestock systems contribute over $1.4 trillion in global assets and this is expected to double in the next 20 years (Alders, 2021; Tucker et al., 2013). Historically, economics have driven the decision-making process in swine production, and will continue to have a fundamental role to maintain economic viability/sustainability. Pressures placed on the swine industry to enhance sustainability practices across production are placing new pressures on the economic sustainability of the industry. Some advances that yield more “sustainable production” may have positive economic benefits, but many simply add costs. An increased expense without a concomitant increase in yield creates new economic pressures on the system. If input increases without an increase in yield, the only method to maintain or achieve economic sustainability is to increase the price of the product. Publicly, we discuss the importance of sustainability, but are we willing to pay to achieve sustainability?

Increasing public pressure for the industry to become more environmentally sustainable and change management practices to be more societally appealing are often applied without acknowledgment of cost and consequently the potentially negative impact on the sustainability of pork production. This phenomenon has been highlighted by researchers who reported consumers' desire for change, but their unwillingness to pay the cost of change in the form of a higher priced product (Von Keyerslingk, 2015; Yeh, 2021). Economic sustainability is further complicated by the global nature of agriculture. Country-specific, or even region-specific changes in pig rearing that increase environmental sustainability and/or pig health and welfare may not be economically sustainable if changes have unmitigated costs and are not globally implemented, because consumers may opt to buy less expensive imported pork. The economic impact of being “sustainable” from an environmental, societal, and animal health and well-being perspective must be considered. Ultimately, pork production is not sustainable in the long term if the costs are too high, and consumers are unwilling to pay. On the flipside, for pork production to remain sustainable we cannot ignore things like environmental footprint, welfare, and the risk of zoonotic disease simply because there is a cost. The question becomes what immediate long term monetary cost is the consumer willing to pay for the long-term benefits of sustainability? And will their willingness increase with an education of the big picture gains associated with sustainability programs.

## Social Sustainability

Modernization of production animal practices coupled with production of a safe, nutritious, and plentiful source of animal protein is directly linked to our ability to feed the world’s growing population and for that population to take a closer look at production animal agriculture. Leading to public identification of both real and perceived issues within animal agriculture and swine production that now must be addressed to satisfy societal concerns. Specifically, the public has directed its attention to animal welfare, adding animal welfare directly to many definitions of sustainability. Growing public concern for farm animal welfare has directly impacted husbandry practices including how food animals are housed and cared for (Thompson, 2011). Modern concerns can be categorized into three major categories:

1. physical functioning: the animal should be able to function and thrive without hindrance or pushed to the point of mental of physical failure,
2. naturalness: the expression of natural behavior related to the species, and the
3. subjective interactions: the animal can experience positive states with reduced negative states (fear) within its environment (Fraser, 2008b).

In the last four decades, there has been a significant increase in the global consumption of animal products (Steinfeld, 2006). Increased population growth over the next decade will contribute to further increases in animal product consumption, particularly in countries with higher per capita income (Food and Agriculture Organization of the United Nations, 2022; Thornton, 2010). Global animal agriculture systems have also seen an intensification of production systems to meet growing demands for animal protein. (Tucker et al., 2013). However, the increase in intensification of production coupled with growing concerns about animal welfare, the environment, and the ethics of animal production practices have polarized public opinion toward production systems (Fraser, 2008a; The World Bank, 2009; Croney and Anthony, 2010; Croney et al., 2012; Henchion, 2022). This polarization has resulted in greater regulation of production practices to appease outspoken critics of animal agriculture, culminating in the passage of legislature like Proposition 12 in the state of California that will indelibly impact food animal production and sale of animal protein within California (Bursey, 2018), but also has wider impacts due to restrictions on imported products as well. The topic of animal welfare, as it pertains to sustainability in food animal systems, is a complex issue that is driven by consumer attitudes towards animal husbandry practices. Broom (2010) claims “no [livestock] system can be sustainable if a substantial proportion of people finds aspects of [production] now, or of its consequences in the future, morally unacceptable”. In response to public attitude towards food animal systems, the United Nations Committee on World Food Security (UN-CFS) incorporated improved food animal welfare as part of its draft recommendations for sustainable agricultural development (Legg, 2017).

Increased public attention on space allocated per animal in a production system has required production houses to increase cage and pen space per animal leading to partial or complete redesign of facilities subsequently increasing financial loss both from infrastructure redesign and reduction in animals per square meter (Sumner et al., 2011; Xin et al., 2011). California proposition 12, which requires in- and out-of-state farmers to increase housing space for egg-laying hens, breeding pigs, and calves raised for veal is one example of public perceptions impacting food animal agriculture (Hendershot, 2022). To buffer the financial loss, costs for alternatively produced animal products increase. However, consumer behavior and opinion regarding animal welfare and sustainability are minimally reflected in their purchasing patterns and often fail to correspond to their vocalized opinion on the topics (Tucker et al., 2013).

## Swine Health and Well-being

The ever-increasing growth, intensification, and simultaneous concentration of swine production has created novel porcine health management and public health challenges, all of which may impact the sustainability of swine production (Maes et al., 2019). The vast majority of porcine health challenges stem from the large numbers of individual animals amassed in a limited area; this concentration of animal numbers creates opportunities for pathogens to proliferate (Martinez, 2002). The risks of viral and bacterial proliferation are compounded by environmental or physiological stressors (such as heat stress or weaning) that can depress the immune system (Lalles et al., 2007; Gabler and Pearce, 2015). Existing disease challenges are exacerbated by increasing levels of antibiotic resistance (Mathew et al., 1999). Bacterial diseases that were once easily treated are more challenging to medically manage when antibiotics are not available or effective (Mathew et al., 1999; Muurinen et al., 2021). Finally, human population growth impacts swine health management. The growing population has created increased demand for pork, thus increasing the global pig herd. Additionally, increased numbers of humans are geographically, if not physically, interacting with livestock and wildlife, magnifying the risks of zoonotic disease transmission (Davies, 2012).

The pathogens that most adversely impact the overall sustainability of intensive swine production are viral agents such as Porcine Epidemic Diarrhea Virus (PEDv), Porcine Reproductive and Respiratory Syndrome virus (PRRSv), African Swine Fever (ASF), and Foot and Mouth Disease (FMD) (VanderWaal and Deen, 2018). These agents are highly contagious and are responsible for billions of dollars of industry losses (Morgan and Prakash, 2006; Nieuwenhuis et al., 2012; Schulz and Tonsor, 2015; Mason-D’Croz et al., 2020; Brown et al., 2021; Shi, 2021). There are no definitive treatments for these viral pathogens; their spread is controlled with intense biosecurity and aggressive quarantine and culling practices. Vaccination would be highly beneficial to further control these viral challenges, but thus far, actual vaccination has been met with limited success as many of these agents tend to mutate (Zhou et al., 2021). Additionally, even if these infections are not fatal by themselves, they tend to depress the immune system of pigs, leaving them extremely vulnerable to bacterial co-infections, many of which are devastating (Saade et al., 2020).

Porcine Epidemic Diarrhea (PED) emerged in North American swine production in 2013. Mortality rates were close to 100% in neonatal piglets, and the disease caused significant production losses in older pigs (Junget al., 2020). Now, PED can be reasonably controlled with close monitoring and aggressive induction of maternal immunity that is sufficiently protective of neonatal piglets. The emergence of this pathogen prompted the development of hitherto unused biosecurity measures, including air filtration, truck washing, and feed surveillance (Jung and Saif, 2015; Jung et al., 2020).

Porcine Reproductive and Respiratory Syndrome virus is a consistent scourge of North American swine production, resulting in reproductive losses in sows and production losses from respiratory morbidities and fever in growing swine. Though there is a vaccine for PRRSv, the rapid mutation of this virus from non- to hyper-pathogenic variants has made vaccination risky and potentially ineffective (Lunney et al., 2010; Zhou et al., 2021). Farms try to control PRRSv with a combination of vaccination and biosecurity, but the lack of consistently effective vaccines and easy transmission of the virus make it difficult to control, particularly in regions of greater swine density, and the most economically devastating swine pathogen in the United States (Arruda et al., 2019). The simple fact that the risk of PRRSv increases with swine density makes it a particular challenge for sustainability of current swine production (Arruda et al., 2019).

African Swine Fever (ASF) recently emerged as a global food security concern after devastating Chinese swine production. ASF is a hemorrhagic disease with an almost 100% mortality rate in infected domestic swine. This virus is also extremely difficult to control because it can cause asymptomatic infections that result in carrier animals that can infect new herds, it can live in a tick vector for over 5 years, and is otherwise extremely difficult to definitively diagnose. Efforts to develop a safe and effective vaccine have been unsuccessful. Thus far the only means of controlling ASF are aggressive culling measures and biosecurity (Turlewicz-Podbielska et al., 2021). ASF holds the greatest threat to the potential sustainability of global swine production and will remain so until an effective control method is developed.

Bacterial agents pose different challenges to swine health and management. Though some of these diseases can be definitively treated with antibiotics, increasing rates of antibiotic resistance, limited capacity for appropriate culture and sensitivity diagnostics, the rapid pathogenesis of many respiratory infections, and limited availability of some antibiotics for use in livestock can make management of these diseases extremely challenging (Sade, 2020). Additionally, some agents such as *Streptococcus suis* and *Mycoplasmaspp.* often fail to respond to antibiotic treatment (Feng et al., 2014; Saade et al., 2020).

Increasing rates of antibiotic resistance have been observed in manure collected from swine production facilities (He et al., 2016; Pholwat et al., 2020; Monger et al., 2021). Fears about increased rates of observed antibiotic resistance in animal agriculture prompted regulatory agencies to limit “medically important” antibiotic use in livestock to “prevent and treat” disease (Monger et al., 2021). This has brought antimicrobial use under the discretion of the attending veterinarian; antimicrobials are no longer allowed to be used to improve growth or feed efficiency (Wallinga et al., 2022). Nevertheless, there are additional calls to further limit the use of antibiotics in livestock. The World Health Organization describes the antimicrobial resistance to be among the “biggest threats to global health, food security and development” (Wallinga et al., 2022). This crisis is compounded by a lack of novel antibiotics in development for animal and human use. However, the use of antibiotics in livestock remains a balance between the public health risk of generating antibiotic resistant bacteria and maintaining animal health and welfare (Wallinga et al., 2022).

Bacterial pathogens represent zoonotic and food safety challenges to the sustainability of swine production. Salmonellosis is a frequent cause of human infection, though most studies have found that pork is a less-common source of contamination than other proteins (Guo et al., 2011; Uddin Khan et al., 2013). However, salmonella remains a significant source of morbidity and mortality in swine production in different parts of the world, and approximately 50% of U.S. swine operations test positive for Salmonella. Discouragingly, 20% of swine-derived salmonella infections are also multidrug resistant, making it a major zoonotic pathogen of concern (Bearson, 2022).

*E. coli* O157:H7 is an additional important zoonotic pathogen; infection can result in diarrhea, hemorrhagic colitis, and hemolytic-uremic syndrome in humans and pigs that can be passed via contamination or fecal-oral contact (Smith and Huggins, 1983; Tarr, 1995). In a recent study examining antibiotic resistance in *E. coli* isolated from swine manure, 91.5% of the isolates were multi-drug resistant (Wang et al., 2021). *E. coli*, particularly drug resistant *E. coli*, is a considerable public health concern because of environmental application of swine manure which may result in contamination of water sources, feed, and other agriculture which may result in human infection (Anderson and Sobsey, 2006; Jakobsen et al., 2010).

One strategy to mitigate the use of antibiotics, and therefore the selection for antibiotic resistant bacteria, in livestock has been the explosive growth of the development of antibiotic alternatives. Though the majority of the development of antibiotic alternatives has focused on reproducing the growth and feed-conversion effects of subtherapeutic levels of antibiotics by modulating the immune system or improving capacity to extract nutrients from feed, the focus of other alternatives has been to specifically reduce viral or bacterial burden. Unfortunately, the efficacy of these alternatives has been inconsistent. This inconsistency makes most antibiotic alternatives not viable replacements for antibiotics in the health management of the swine herd (Kurt et al., 2019). Additional work is being done in this field, but investigators have had limited success thus far.

Beyond the general risk of antimicrobial resistant bacteria being generated from antibiotic use in intensively managed swine production systems, swine are a source of multiple zoonotic viral pathogens that have the potential to be severe public health issues (Uddin Khan et al., 2013). One of the primary pathogens of concern when considering the human–porcine interface is influenza (Kothalawala et al., 2006). Swine influenza caused a pandemic in humans in 2009, and has remained an economically important pathogen in swine production as it tends to result in bacterial co-infections that exacerbate the severity of infections (Ma, 2020). Unfortunately, swine and humans can pass influenza between species, though the human–human transmission of the virus tends to be limited with most serotypes (Anderson et al., 2021; Ma, 2020). Influenza is difficult to control in swine and human populations because of the immense variability and tendency to mutate across time and in geographic locations (Ma, 2020). Additionally, the near-100% morbidity of the virus is compounded by global movement of pigs and people, resulting in rapid spread (Ma, 2020).

Additional zoonotic viral pathogens that are far more limited in geographical localization, but potentially more devastating are Ebola and Nipah viruses. Ebola-Zaire virus causes hemorrhagic fever and can result in 40%–100% mortality in humans (Uddin Khan et al., 2013; Haddock et al., 2021). Pigs are susceptible to and can transmit Ebola-Zaire and Ebola-Reston to nonhuman primates, and humans have been infected by Ebola-Reston virus, indicating that pigs may play a role in future Ebola outbreaks (Marsh, 2011; Uddin Khan et al., 2013). Nipah virus was identified in Malaysia and Singapore where porcine infection resulted from contact with wild bats. The virus spread rapidly among swine farms due to producers attempting to market sick pigs. Unfortunately, the virus spread to humans in contact with sick pigs, resulting in approximately 40% mortality (Uddin Khan et al., 2013). Increased interaction between humans, livestock, and wildlife will result in the emergence of additional zoonotic viral pathogens that may prove devastating to swine and human health. The only means to control such outbreaks is with biosecurity and aggressive quarantine measures.

Unfortunately, the exponential growth of both the human population and global swine herd complicates the control and eradication of pathogens that adversely impact production and pose threats to public health. Niche markets specializing in different swine breeds or specifically raised swine on small farms make disease surveillance challenging (as was observed with the spread of Nipah Virus in Malaysia and Singapore) (Uddin Kahn et al, 2013). Additionally, public pushback on disease control efforts like culling and quarantine further limit the feasibility for agencies to control outbreaks (Lederman, 2016). Future work needs to focus on controlling physiologic and social stressors in swine production systems to minimize immunosuppression and maximize the ability for animals to resist infection. Other work should focus on improving biosecurity, particularly in regions of the world where humans and animals are in close contact with wildlife to prevent viral and bacterial spillover. The ability of the swine industry to manage disease pressures is the most significant facet of maintaining sustainability of production; animals will not grow if they are ill, people will not consume pork if it is not safe, and pigs cannot be marketed if they are dead.

## Conclusions

**Figure 3.**
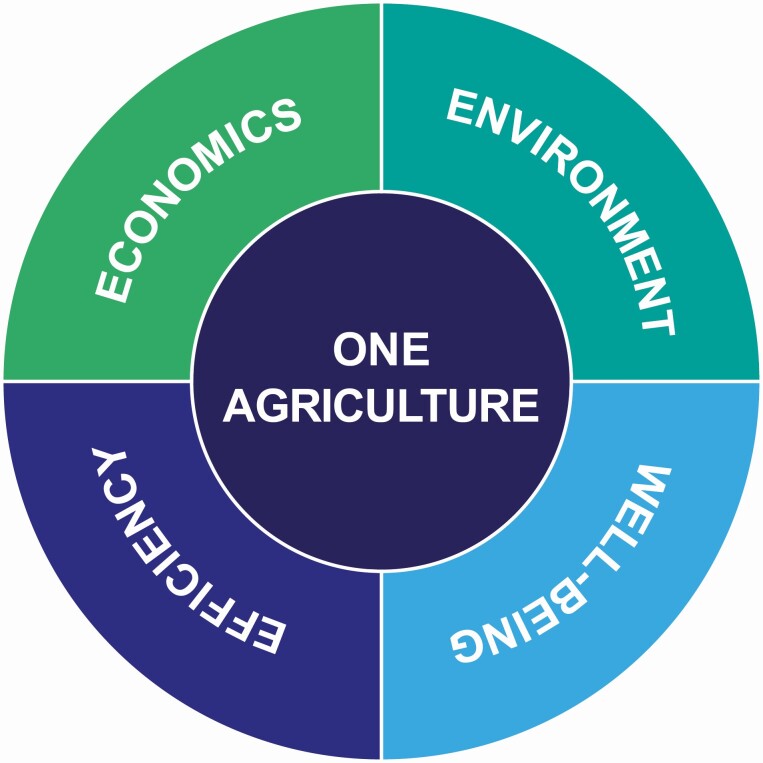
One-agriculture.

One major challenge when discussing sustainability is deciding where the box needs to be drawn with regards to what pieces need to be included to look at the whole of sustainability. For example, it would be easy to draw the box around a pig and only look at the direct outputs of the pig, but production of that pig requires crops to be grown, harvested, and processed to make feed, for the pig. The feedmill, that manufactures the feed uses energy to run grinding and mixing equipment. The feedmill had to be constructed, which used energy and raw building materials. The concrete floor of the feedmill had to be trucked in. The cement trucks had to be manufactured, and the truck manufacturing plant uses energy. So the box can get to be very large. The larger the box gets, the more complex the sustainability question becomes. Ultimately, we need to sustain the planet and swine production has to be part of that process (Figure 3). Therefore, while it is important to strive towards sustainability in swine production, ultimately swine production is only one piece of agriculture and agriculture is one piece of the planetary sustainability puzzle. It will take a coordinated effort across all facets of society to truly move the sustainability needle. Individual pieces like environmental sustainability of swine production should continue to be advance, but it is important to understand the impacts of progress in one area on the whole of sustainability.

So, is sustainability sustainable? Well, we all hope so, but there are a finite number of natural resources available and with a still growing human population, it will be increasingly more challenging, and let’s face it, we have not done the best job so far! We need to shift away from placing blame on others or drawing the box to bias sustainability efforts or to effectively blame one area over another and focus more on fitting all the pieces together for a sustainable planet.

*Conflict of interest statement*. None declared.

## About the Authors

**Caitlin Vonderohe.** Caitlin Vonderohe is an Instructor at the USDA-ARS Children’s Nutrition Research Center (CNRC) in the Department of Pediatrics: Section of Gastroenterology, Hepatology and Nutrition at Baylor College of Medicine. She received her B.S. at Purdue University, her M.S. as Texas A&M University, and dual Ph.D. (Animal Science: Non-ruminant nutrition) and D.V.M. at Purdue University. She completed her postdoctoral fellowship in pediatric nutrition at the CNRC under the direction of Dr. Doug Burrin. Dr. Vonderohe’s current research focuses on using the pig as a model to study gastrointestinal and liver diseases that impact term and preterm infants. She is interested in exploring translational, nutritional and pharmaceutical approaches that will improve clinical outcomes in pediatric patients.

**Lauren A. Brizgys.** Lauren Brizgys is a third year PhD candidate in the Department of Animal Sciences at Purdue University. She received her B.S, M.S, and Graduate Certificate (M.S) from Purdue University’s College of Agriculture and College of Education where she studied Animal Science, Agricultural Science Education and Communication, and Learning/Curriculum Design. Lauren Brizgys’ current research focuses on eliminating or reducing short- and long-term, transport-induced reductions in feed efficiency and growth through increased amino acid supplementation at varying stages of swine production pre- and post-weaning. Lauren is broadly interested in nutritional intervention strategies to reduce oxidative stress during production, improving immune vigor during stress, and endocrinological responses to nutritional interventions during a stress challenge.

**Jacob Richert.** Jacob Richert is a PhD candidate in the Department of Animal Science at Purdue University in swine nutrition. Jacob received his B.S. from Purdue University and his M.S. in Swine Nutrition from Kansas State University. Jacob’s PhD dissertation work is focused on the nursery age pig. His main interests are how nutrition and weaning age can affect gut development and pig health during this critical weaning period, ultimately impacting pig growth performance. Jacob is interested in finding new ways to feed the weanling pig and creating an optimal way to transition piglets so they may flourish post-weaning and be positioned to achieve their best possible lifetime performance.

**John “Scott” Radcliffe.** Scott Radcliffe is a Professor and Assistant Head in the Department of Animal Sciences at Purdue University. He received his B.S., M.S. and Ph.D. from Virginia Tech and was briefly on the faculty in the Department of Animal and Poultry Sciences at Virginia Tech prior to moving to Purdue University. Dr. Radcliffe has two fundamental research interests: 1) environmental nutrition—how do we feed pigs to ensure the highest level of environmental sustainability? and 2) gut health. Within those two areas, Dr. Radcliffe’s work can be partitioned into four categories: 1) reduction of the environmental impact of production animal agriculture (primarily swine) through increasing the efficiency of nutrient utilization and reducing nutrient excretion, 2) use of animal models to study nutrient absorption/utilization in human disease states, 3) gut health and function, and 4) the immune response in food allergy development and food-related digestive disorders.
